# KCNQ1OT1 polymorphism rs35622507 and methylation status of KCNQ1OT1 promoter influence the drug resistance to L-OHP

**DOI:** 10.18632/aging.203906

**Published:** 2022-02-22

**Authors:** Caihong Zhang, Yonglin Wang

**Affiliations:** 1 Proctology Department, Xing Yuan Hospital of Yulin, Yulin 719000, Shaanxi, China; 2 Pharmacy Department, Yangling Demonstration Zone Hospital, Xianyang 712100, Shaanxi, China

**Keywords:** colon cancer, oxaliplatin resistance, KCNQ1OT1, rs35622507, miR-34a

## Abstract

Background: LncRNA potassium voltage-gated channel subfamily Q member 1 opposite strand/antisense transcript 1 (KCNQ1OT1) has been reported to promote resistance to chemotherapy in colon cancer by inhibiting the expression of miR-34a. And the methylation of KCNQ1OT1 was also reported in the pathogenesis of various diseases. In this study, we aimed to study the combined effect of allele variation of KCNQ1OT1 polymorphism rs35622507 and methylation status of KCNQ1OT1 promoter in the treatment of colon cancer.

Methods: The expression levels of KCNQ1OT1, miR-34a, and ATG4B mRNA were assessed by qRT-PCR. ATG4B protein expression was analyzed by Western blot analysis. TUNEL and MTT assay were performed to examine the cell apoptosis and viability. Luciferase assays revealed the relationship between KCNQ1OT1, miR-34a and ATG4B.

Results: Carrier of allele 10 and methylated promoter in KCNQ1OT1 was associated with decreased KCNQ1OT1/ATG4B expression, increased miR-34a expression and enhanced apoptosis in colon cancer tissue samples. And subsequent luciferase assay showed that miR-34a could bind to KCNQ1OT1 and ATG4B at specific binding sites. The knockdown of KCNQ1OT1 significantly suppressed the KCNQ1OT1/ATG4B expression, improved the miR-34a expression and reduced the viability of HCT116 and SW480 cells. The over-expression of ATG4B notably restored the cell viability loss and apoptosis increase induced by the knockdown of KCNQ1OT1. Moreover, oxaliplatin (L-OHP) treatment elevated the apoptosis of HCT116 and SW480 cells.

Conclusions: The drug resistance in the treatment of colon cancer is most reduced in patients carrying allele 10 and methylated in KCNQ1OT1 promoter. This function is accomplished by the signaling pathway of KCNQ1OT1/miR-34a/ATG4B.

## INTRODUCTION

Globally, colorectal cancer (CRC) is one of the most common cancers and a primary cause of death due to cancer. The majority of patients with CRC will at some point die of this awful illness [1]. The typical radiation treatment routines 5-fluorouracil/Leucovorin/Oxaliplatin (FOLFOX) and XELOX still functions in the treatment of CRC in advanced stages. Oxaliplatin (OXA) is a DNA-alkylating representative and third-generation platinum drug that disrupts DNA replication and transcription and generates reactive oxygen species (ROS) production, promoting apoptosis [2, 3].

Currently, CRC is one of the most frequent causes of fatality due to cancer [1]. Intravascular administration of oxaliplatin (L-OHP), a third-generation diaminocyclohexane platinum substance, has been a standard medicine for both resectable and unresectable CRC in the clinic [4–6]. In approximately 40-45% of CRCs, a Kirsten rat sarcoma virus gene (KRAS) anomaly is observed that reduces the efficacy of L-OHP [7]. Chemoresistance poses the key challenge to the successful treatment of cancer patients. The most common mechanisms associated with cancer resistance to chemotherapy include upregulation of ATP-dependent efflux pumps, drug metabolizing enzymes, and DNA repair systems [8, 9].

LncRNAs are a specific class of non-protein-coding transcripts with greater than 200 nucleotides. LncRNAs were at first considered as transcriptional noise, nevertheless, arising proof currently suggests that lncRNAs perform various functions, such as epigenetic regulation and gene transcription [10, 11]. MicroRNAs (miRNAs) belong to a class of small, single-stranded noncoding RNAs associated with the regulation of different biological processes, such as advancement, spreading, survival, inflammation, as well as tumorigenesis [12]. LncRNAs are well-known to function in various intracellular pathways using different mechanisms including the sponging of miRNA as decoys [13]. Specifically, recently studied ceRNAs are a crucial part of gene expression regulation by lncRNA [13]. Here, the ability of lncRNA, KCNQ1OT1, to potentially sponge the miR-34a has been reported, in turn regulating the cysteine protease enzyme, ATG4B leading to autophagy. The miRNAs are endogenous noncoding RNAs, which function as necessary post-transcriptional regulators of gene expression [14, 15]. They bind specifically to 3’-untranslated region (3'-UTR) site of mRNA, in turn inhibiting the translation and/or mRNA degradation [16]. Based on previous reports, the miR-34a could target ATG4B directly and decrease autophagy [17, 18]. In this paper, we validated that along with miR-34a, miR-34c-5p, also a member of miR-34 family, could specifically target ATG4B and suppress rapamycin induced-autophagy by degrading ATG4B mRNA.

The resistance developed by the CRC cells to L-OHP chemotherapy has been studied thoroughly. The studies showed the up-regulation of lncRNA KCNQ1OT1 in tumor cells in contrast to the healthy tissues. In addition, it was shown that ATG4B, a key enzyme, was up-regulated by KCNQ1OT1 which further induced protective autophagy. These data highlight role of KCNQ1OT1 in developing chemoresistance to L-OHP resulting from the sponging of the miR-34a and ATG4B-induced protective autophagy increase. Functional assays show a considerable relationship between genotype and phenotype in which the STR polymorphism genotypes produce decreased KCNQ1OT1 expression and enhanced CDKN1C expression. Computational analyses suggest that rs35622507 might obstruct the KCNQ1OT1- folding structure and influence its expression through a structure-dependent mechanism. For that reason, the unique STR polymorphism may serve as a potential marker for hereditary sensitivity to HCC.

LncRNA KCNQ1OT1 has been reported to promote the chemoresistance of colon cancer by inhibiting the expression of downstream miRNA miR-34a [19]. The methylation of KCNQ1OT1 was also reported to be involved in the pathogenesis of various diseases [20–22]. In this study, we examined the combined outcome of allele variation of the KCNQ1OT1 polymorphism rs35622507 and methylation status of KCNQ1OT1 promoter on the drug resistance in the treatment of colon cancer and its underlying molecular mechanisms.

## MATERIALS AND METHODS

### Human subjects sample collection

In this study, we recruited colon cancer patients and grouped them according to the results of genotyping of the KCNQ1OT1 polymorphism rs35622507 by TaqMan and methylation evaluation of the KCNQ1OT1 promoter region. All participants were divided into four groups: Group A: carrier of allele 10 + methylated KCNQ1OT1, Group B: carrier of allele 10 + non-methylated KCNQ1OT1, Group C: non-carrier of allele 10 + methylated KCNQ1OT1, and Group D: non-carrier of allele 10 + non-methylated KCNQ1OT1. The characteristics of the patients, including sex, age, tumour size and tumour stage, were collected. Cancer tissue samples were collected for subsequent analysis. The methylation profiles of KCNQ1OT1 promoter region were determined by bisulfite mutagenesis and sequencing according to previous published methods in the cancer tissue samples collected [23]. The Institutional Ethics Committee of Yangling Demonstration Zone Hospital approved the protocol of this study (Approval ID: SXYLSFQYY083-996). Signed written informed consent was obtained from each participant before the initiation of this study.

### DNA extraction and genotyping

A DNA filtration set (Qiagen, Germantown, MD, USA) was used to isolate genomic DNA from collected peripheral blood samples. DNA pieces including rs35622507 were intensified with dual primers (forward primer: 5’ -GCTGGCTGTTTCTATTCAGTG-3’, reverse primer: 5’- GGTCCTAGCACCTGGTTTGAC-3’) bought from Sangon Biotech (Shanghai, China). The polymerase chain reaction (PCR) products were examined by 7% nondenaturing polyacrylamide gel electrophoresis).

### RNA isolation and real-time PCR

Using TRIzol reagent (Invitrogen, Carlsbad, CA, USA), RNA extraction from tissues or cells was carried out. Takara reverse transcription system (Dalian, China) was utilized to reverse transcribe the RNA into cDNA. qPCR analysis was performed with Applied Biosystems 7500 Real Time PCR System (Thermo Fisher Scientific, Waltham, MA, USA) using iQ™ SYBR^®^ Green Supermix Kit (Bio-Rad, Hercules, CA, USA). KCNQ1OT1, miR-34a, and ATG4B primers were purchased from Sangon Biotech (Shanghai, China). To calculate the gene expression, the 2^− ΔΔCt^ method was used.

### Cell culture and transfection

The human CRC cell lines HCT116 and SW480 were obtained from American Type Culture Collection (Manassas, VA, USA). Both cell lines were preserved in RPMI-1640 medium supplied with 10% fetal bovine serum (FBS) (Thermo Fisher Scientific, Waltham, MA, USA), and incubated at 37° C with 5% CO_2_. Model 1 included HCT116 and SW480 cells divided right in 2 groups, i.e., 1. NC siRNA group (HCT116 and SW480 cells both transfected with NC siRNA) and 2. si KCNQ1OT1 group (HCT116 and SW480 cells both transfected with KCNQ1OT1 siRNA), Model 2 included three groups of HCT116 and SW480 cells: 1. NC siRNA (HCT116 and SW480 cells both transfected with NC siRNA), 2. KCNQ1OT1 siRNA group (HCT116 and SW480 cells both transfected with KCNQ1OT1 siRNA), and 3. KCNQ1OT1 siRNA + pcDNA-ATG4B group (HCT116 and SW480 cells both transfected with KCNQ1OT1 siRNA plus the pcDNA vector carrying ATG4B). Model 3 included six groups of HCT116 as well as SW480 cells: 1. NC siRNA group (HCT116 and SW480 cells both transfected with NC siRNA) 2. si KCNQ1OT1 group (HCT116 and SW480 cells both transfected with KCNQ1OT1 siRNA), 3. KCNQ1OT1 siRNA + pcDNA-ATG4B group (HCT116 and SW480 cells both transfected with KCNQ1OT1 siRNA plus the pcDNA vector carrying ATG4B), 4. 1. NC siRNA group treated with L-OHP (HCT116 plus SW480 cells both transfected with NC siRNA and treated with 10 μM/L L-OHP) 5. si KCNQ1OT1 group treated with L-OHP (HCT116 and SW480 cells both transfected with KCNQ1OT1 siRNA and treated with 10 μM/L L-OHP), 6. KCNQ1OT1 siRNA + pcDNA-ATG4B group treated with L-OHP (HCT116 and SW480 cells both transfected with KCNQ1OT1 siRNA plus the pcDNA vector carrying ATG4B and treated with 10 μM/L L-OHP). As per the manufacturer's protocols, the HCT116 cells were transfected using Lipofectamine^®^ 2000 (Thermo Fisher Scientific, Waltham, MA, USA), while the SW480 cells were transfected with HiPerFect (Qiagen, Germantown, MD, USA).

### Cell proliferation assay

After transfecting HCT116 and SW480 cells with negative control siRNA, siRNA KCNQ1OT1, or siRNA KCNQ1OT1 combined with a pcDNA vector carrying ATG4B, the standard MTT staining protocol was performed. After a 24- or 72-hr incubation, MTT fluid was added into each well, and the plate was cultured at 37° C for an additional 4 hrs. Afterwards, the plate was aspirated, and the wells were washed with PBS and permitted to dry for approximately 2 hrs. Additionally, 200 μl of DMSO was pipetted into each well. Using ELX800 UV global microplate viewers (Bio-Tek Instruments, San Jose, CA, USA) with a reference wavelength of 630 nm, the absorbance was established spectrophotometrically at 570 nm.

### Luciferase assay

Using luciferase reporter assays, the regulatory relationships among KCNQ1OT1 and miR-34a and among miR-34a and the 3′-UTR of ATG4B mRNA were established. PCR was performed using PDP to amplify KCNQ1OT1 cDNA and the 3′-UTR of ATG4B cDNA carrying the expected binding site of miR-34a. After approximately 48 h of transfection of miR034a or miRNA-NC into CRC cells, a luciferase assay was performed utilizing a luciferase assay kit (Thermo Fisher Scientific, Waltham, MA, USA).

### Western blot analysis

The primary antibody used was anti-ATG4B (Abcam, Cambridge, MA, USA). Protein was isolated from HCT116 and SW480 cells using RIPA buffer (Abcam, Cambridge, MA, USA), and the bicinchoninic acid protein assay kit (Thermo Fisher Scientific, Waltham, MA, USA) was used to determine the protein concentration.

### Apoptosis analysis

The HCT116 or SW480 cells, which were carefully transfected with siNC or siKCNQ1OT1 siRNA or pcDNA carrying ATG4B with or without L-OHP treatment for 36 h, and then at 37° C, it digested and incubated with Annexin V and propidium iodide (Thermo Fisher Scientific, Waltham, MA, USA) for 30 min. Using Flowing Software, the apoptotic fractions of the cells were analyzed.

### Immunohistochemistry

Tissue microarray blocks were used for immunohistochemistry staining. Briefly, the TMA blocks were immersed in 10 mM EDTA buffer and reacted at 125° C for 10 min to retrieve the ATG4B antigen. After 3% H2O2 in MeOH treatment and 3% BSA treatment, the slides were incubated with antibodies against ATG4B (dilution 1:100; Cell Signaling Technology, Danvers, MA, USA) in cold temperature for one day. IHC staining for the ATG4B protein was performed at room temperature, and counterstaining with haematoxylin was performed.

### TUNEL assay

After thawing the frozen samples, they were stained using a TUNEL kit (Abcam, Cambridge, MA, USA). Later, the thawed samples were dipped in a 3% hydrogen peroxide solution at room temperature for approximately 10 min and subsequently washed with PBS. Next, 50 μL of proteinase K solution was added, and the tissue proteins were removed by hydrolysis at room temperature for 20 min. The antigen was retrieved. Then,50 μL of a TdT enzyme solution was pipetted onto the slides, and the slides were incubated. Eventually, a few drops of DAPI solution were pipetted onto the slides, and the slides were incubated for a few min prior to observing them under a fluorescence microscope. Cells containing green nuclei were deemed apoptotic cells, while normal cells showed blue nuclei.

### Statistical analysis

All results are reported as the mean ± SD deviation. The statistical relevance of intergroup contrasts was determined by combined Pupil's t-tests. The statistical relevance between multiple groups was determined by one-way ANOVA combined with Tukey’s test as the post hoc test. The statistical analyses were performed using Prism 7.0 software (GraphPad, La Jolla, CA, USA). P = < 0.05 showed statistical relevance.

### Availability of data and material

The data that support the findings of this study are available from the corresponding author upon reasonable request.

## RESULTS

### Differential expression of KCNQ1OT1, miR-34a and ATG4B mRNA in colon cancer tissue samples with distinct genotypes and methylation levels of KCNQ1OT1 promoters

**Figure 1:**
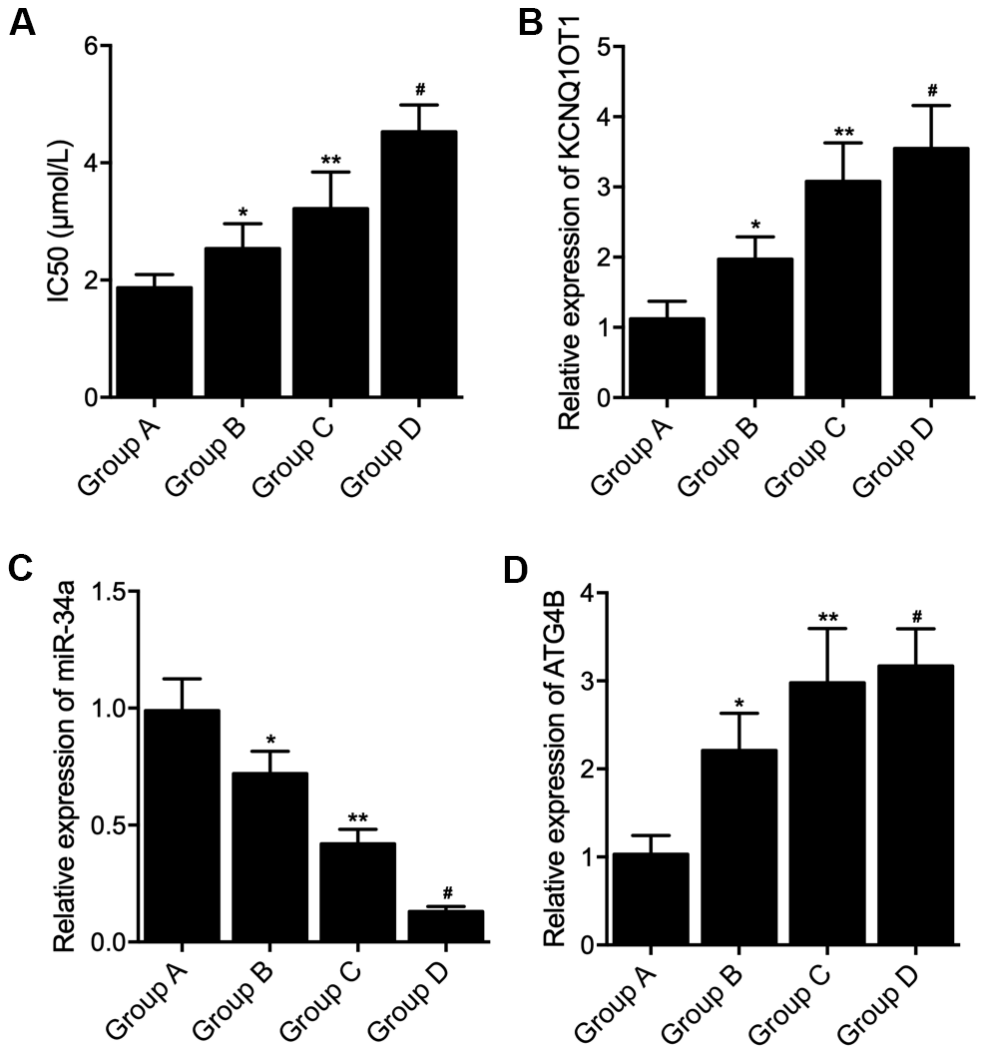
**Differential expression of KCNQ1OT1, miR-34a and ATG4B mRNA in colon cancer tissue samples with distinct genotypes and methylation levels of KCNQ1OT1 promoters.** (**A**) Increased IC50 by L-OHP treatment in Group A, Group B, Group C and Group D (statistical analysis: one-way ANOVA; * P value < 0.05, F value = 8.43 vs. Group A; ** P value < 0.05, F value = 9.52 vs. Group B; # P value < 0.05 F value = 11.22 vs. Group C). (**B**) Increased expression of KCNQ1OT1 in Group A, Group B, Group C and Group D (statistical analysis: one-way ANOVA; * P value < 0.05, F value = 10.96 vs. Group A; ** P value < 0.05, F value =6.37 vs. Group B; # P value < 0.05 F value = 9.58 vs. Group C). (**C**) Decreased expression of miR-34a in Group A, Group B, Group C and Group D (statistical analysis: one-way ANOVA; * P value < 0.05, F value = 5.81 vs. Group A; ** P value < 0.05, F value = 10.99 vs. Group B; # P value < 0.05 F value = 11.95 vs. Group C). (**D**) Increased expression of ATG4B mRNA in Group A, Group B, Group C and Group D (statistical analysis: one-way ANOVA; * P value < 0.05, F value = 9.74 vs. Group A; ** P value < 0.05, F value = 7.27 vs. Group B; # P value < 0.05 F value = 6.39 vs. Group C).

The characteristics of patients including gender, age, tumor size and tumor stage were compared among the four groups, no significant difference were found (Table 1). *Ex vivo* culture of the tumor tissue was performed followed by L-OHP treatment, IC50 was calculated for group A, B, C and D. The IC50 was gradually increased in Groups A, B, C and D (Figure 1A). KCNQ1OT1, miR-34a and ATG4B mRNA expression was examined in the tissue sample collected from patients in the four groups. The expression of KCNQ1OT1 (Figure 1B), as well as the expression of ATG4B mRNA (Figure 1D), was gradually elevated in Groups A, B, C and D (Figure 1B), while the expression of miR-34a was gradually decreased in Groups A, B, C and D (Figure 1C).

**Table 1:** The characteristics of patients recruited in this study.

**Characteristics**	**Carrier of allele 10 + methylated (N=3)**	**Carrier of allele 11 + non-methylated (N=3)**	**Non-carrier of allele 12 + methylated (N=3)**	**Non-carrier of allele 13 + non-methylated (N=3)**	***P* value**
Gender, F/M	1/2	1/2	2/1	1/2	0.780
Age, years					0.364
≥50	1	2	2	1	
<50	2	1	1	2	
Tumor stage (Dukes)					0.508
I, II	2	1	1	2	
III, IV	1	2	2	1	
Tumor size (cm)					0.411
≥3	2	1	2	2	
<3	1	2	1	1	

**Figure 2:**
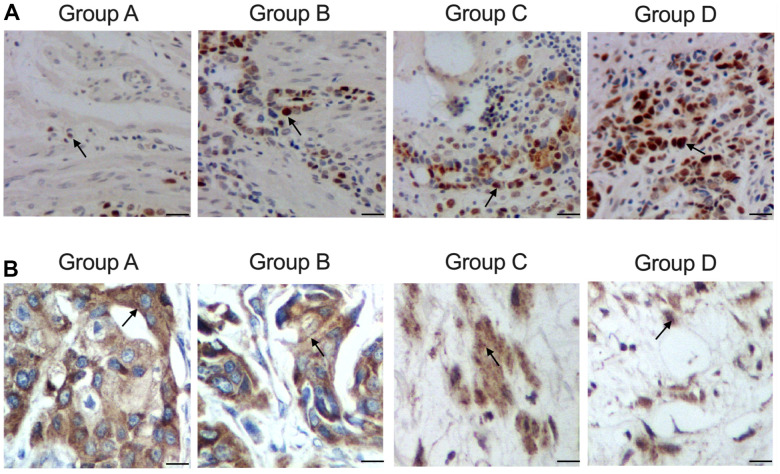
**Distinct level of cellular apoptosis in colon cancer tissue samples with distinct genotypes and methylation levels of KCNQ1OT1 promoters (scale bar: 25 μm).** (**A**) Increased expression of ATG4B protein in group A: carrier of allele 10 + methylated, B: carrier of allele 10 + non-methylated, C: non-carrier of allele 10 + methylated, D: non-carrier of allele 10 + non-methylated. (**B**) Decreased apoptosis in group A: carrier of allele 10 + methylated, B: carrier of allele 10 + non-methylated, C non-carrier of allele 10 + methylated, D: non-carrier of allele 10 + non-methylated.

Furthermore, according to the IHC results while measured the expression of ATG4B protein in the tissue sample collected from the patients, the expression of ATG4B protein was gradually elevated in Groups A, B, C and D (Figure 2A). And TUNEL results which analyzed the apoptosis of the tissue samples collected from the patients indicated that the apoptosis of the tissue samples was gradually decreased in Groups A, B, C and D (Figure 2B).

### The luciferase activities of KCNQ1OT1 and ATG4B were suppressed by miR-34a

**Figure 3:**
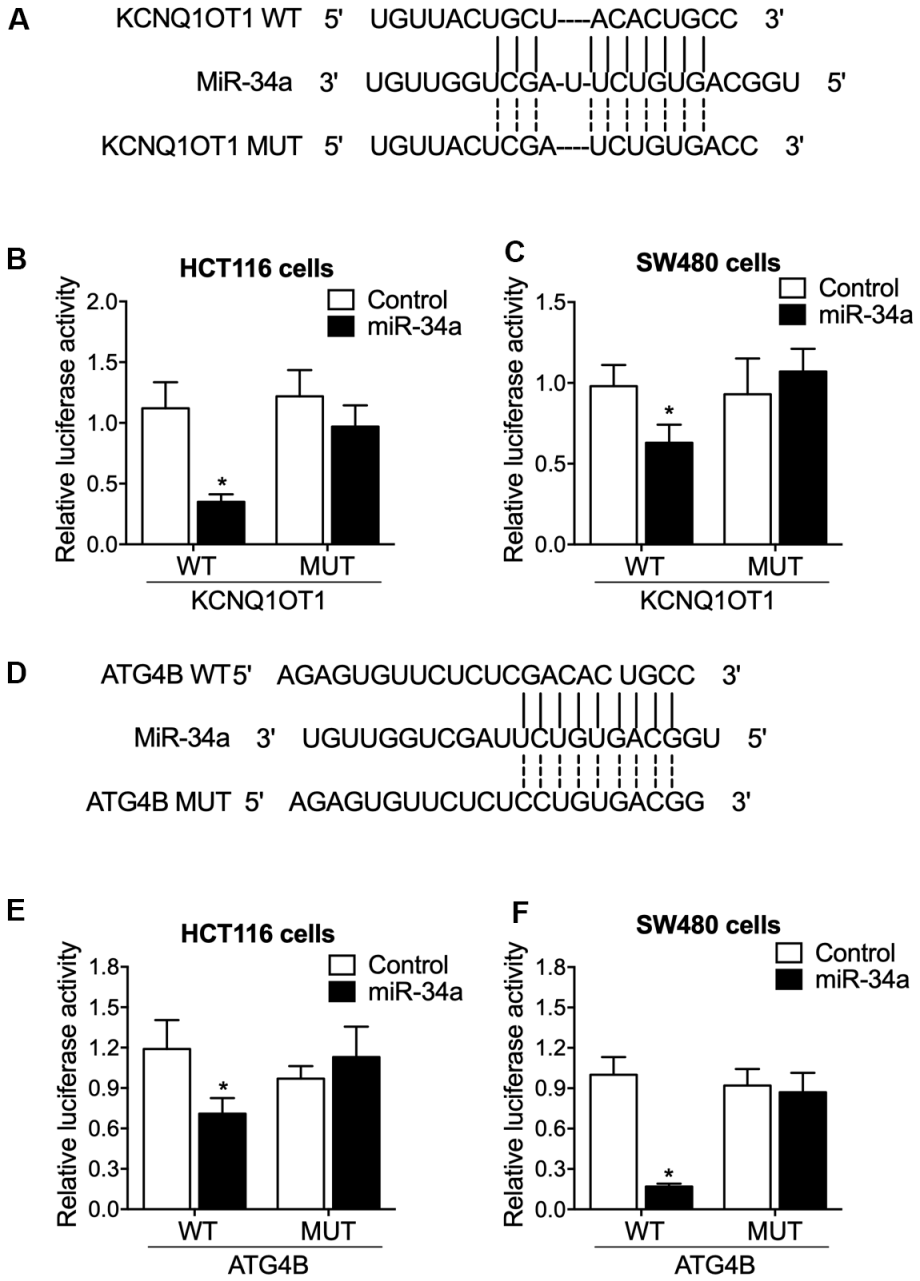
**The luciferase activities of KCNQ1OT1 and ATG4B were repressed by miR-34a.** (**A**) Sequence analysis indicated potential binding of miR-34a to KCNQ1OT1. (**B**) The luciferase activity of KCNQ1OT1 was suppressed by miR-34a in HCT116 cells (statistical analysis: one-way ANOVA; * P value < 0.05, F value = 10.17 vs. control group). (**C**) The luciferase activity of KCNQ1OT1 was suppressed by miR-34a in SW480 cells (statistical analysis: one-way ANOVA; * P value < 0.05, F value = 6.29 vs. control group). (**D**) Sequence analysis indicated potential binding of miR-34a to ATG4B. (**E**) The luciferase activity of ATG4B was suppressed by miR-34a in HCT116 cells (statistical analysis: one-way ANOVA; * P value < 0.05, F value = 7.24 vs. control group). (**F**) The luciferase activity of K ATG4B was suppressed by miR-34a in SW480 cells (statistical analysis: one-way ANOVA; * P value < 0.05, F value = 11.69 vs. control group).

MiR-34a binding sites screening indicated that miR-34a could potentially bind to KCNQ1OT1 (Figure 3A). The luciferase activities of wild-type (WT) KCNQ1OT1 rather than mutant (MUT) KCNQ1OT1 were remarkably reduced by the transfection of miR-34a in HCT116 (Figure 3B) and SW480 cells (Figure 3C). Also, miR-34a binding sites screening indicated that miR-34a could potentially bind to ATG4B (Figure 3D), and the activities of WT ATG4B 3’UTR rather than MUT ATG4B 3’UTR were remarkably reduced by miR-34a in HCT116 (Figure 3E) and SW480 cells (Figure 3F).

### ATG4B effectively restored KCNQ1OT1 siRNA caused cellular viability loss and apoptosis increase

**Figure 4:**
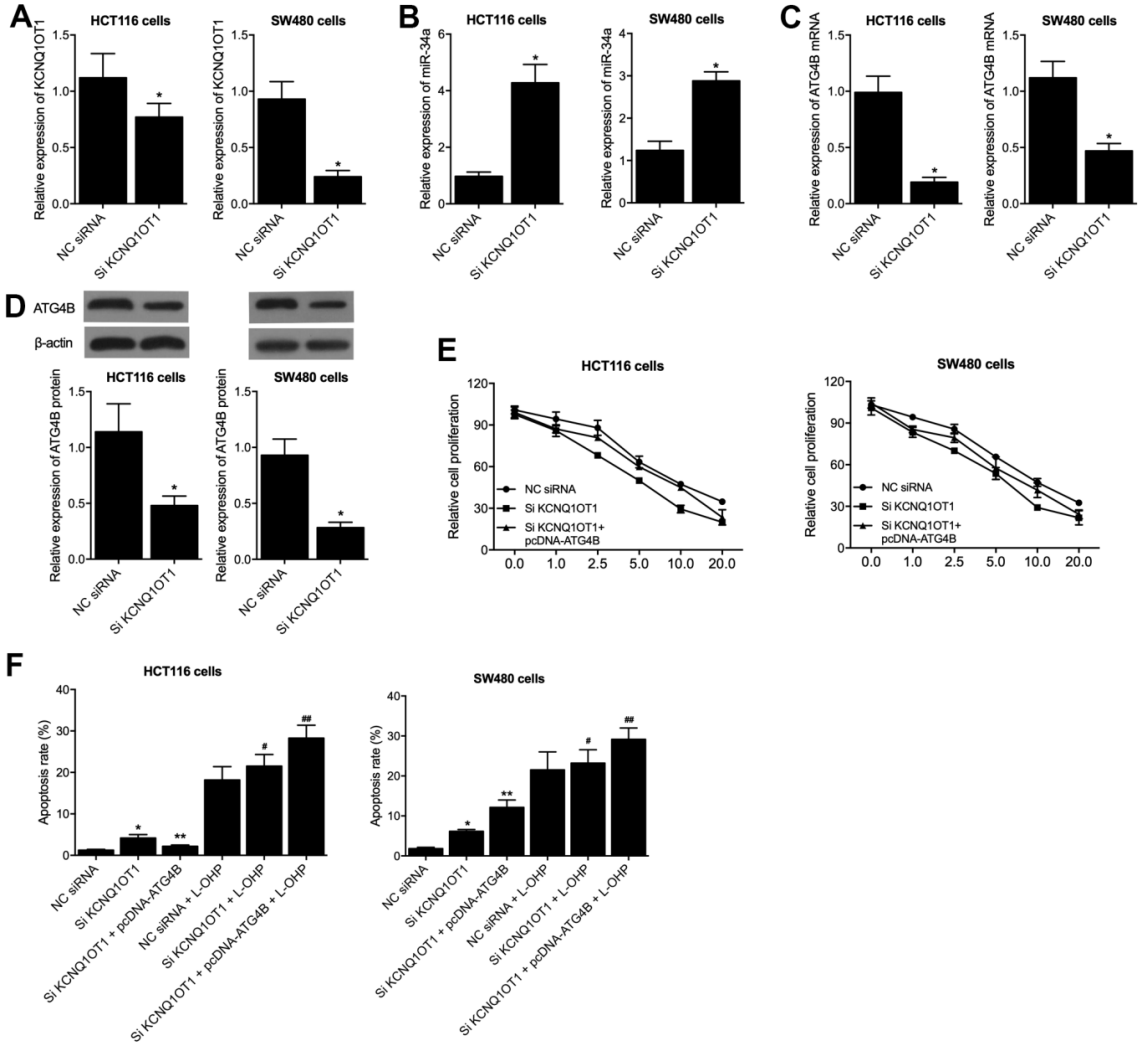
**PcDNA-ATG4B reversed the KCNQ1OT1 siRNA caused dysregulation of HCT116 and SW480 cells.** (**A**) KCNQ1OT1 siRNA suppressed the expression of KCNQ1OT1 in HCT116 and SW480 cells (statistical analysis: Pupil's t-test; * P value < 0.05 vs. NC siRNA). (**B**) KCNQ1OT1 siRNA activated the expression of miR-34a in HCT116 and SW480 cells (statistical analysis: Pupil's t-test; * P value < 0.05 vs. NC siRNA). (**C**) KCNQ1OT1 siRNA suppressed the expression of ATG4B mRNA in HCT116 and SW480 cells (statistical analysis: Pupil's t-test; * P value < 0.05 vs. NC siRNA). (**D**) KCNQ1OT1 siRNA suppressed the expression of ATG4B protein in HCT116 and SW480 cells (statistical analysis: Pupil's t-test; * P value < 0.05 vs. NC siRNA). (**E**) PcDNA-ATG4B reversed the KCNQ1OT1 siRNA caused cell viability decrease in HCT116 and SW480 cells. (**F**) PcDNA-ATG4B reversed the KCNQ1OT1 siRNA caused apoptosis increase in HCT116 and SW480 cells. L-OHP treatment increased the apoptosis level of HCT116 and SW480 cells under distinct conditions (statistical analysis: one-way ANOVA; HCT116: * P value < 0.05, F value = 8.42 vs. NC siRNA; ** P value < 0.05, F value = 7.91 vs. Si KCNQ1OT1; # P value < 0.05, F value = 6.84 vs. NC siRNA + L-OHP; ## P value < 0.05, F value = 11.08 vs. Si KCNQ1OT1 + L-OHP; SW480: * P value < 0.05, F value = 9.99 vs. NC siRNA; ** P value < 0.05, F value = 10.91 vs. Si KCNQ1OT1; # P value < 0.05, F value = 8.79 vs. NC siRNA + L-OHP; ## P value < 0.05, F value = 11.22 vs. Si KCNQ1OT1 + L-OHP).

To gain an insight into the molecular mechanisms of KCNQ1OT1 on colon cancer, KCNQ1OT1 siRNA was transfected into HCT116 and SW480 cells, and the successful transfection of KCNQ1OT1 siRNA was validated by the remarkably suppressed expression of KCNQ1OT1 (Figure 4A). Meanwhile, the expression of miR-34a was remarkably enhanced by KCNQ1OT1 siRNA (Figure 4B), and the expression of ATG4B mRNA and protein was remarkably suppressed by KCNQ1OT1 siRNA (Figure 4C, 4D). Moreover, when co-transfecting pcDNA-ATG4B with KCNQ1OT1 siRNA into the HCT116 and SW480 cells, MTT assay results found that the reduced cell viability induced by knockdown of KCNQ1OT1 was restored by the transfection of pcDNA-ATG4B in HCT116 and SW480 cells (Figure 4E). Furthermore, KCNQ1OT1 siRNA and KCNQ1OT1 siRNA + pcDNA-ATG4B transfected HCT116 and SW480 cells were subjected to L-OHP treatment. Flow cytometry results showed that pcDNA-ATG4B effectively maintained the increased apoptosis rate induced by transfection of KCNQ1OT1 siRNA in HCT116 and SW480 cells. Besides, L-OHP treatments remarkably elevated the cell apoptosis rate of KCNQ1OT1 siRNA and KCNQ1OT1 siRNA + pcDNA-ATG4B cell groups of HCT116 and SW480 cells (Figure 4F).

## DISCUSSION

KCNQ1OT1 is located on human chromosome 11p15.5, a two-domain region regulated by differentially methylated region 1 (DMR1) and KvLQT1 differentially methylated region 1 (KvDMR1), which are two distinct imprinting control regions. DMR1 is methylated on the paternal allele while KvDMR1 is methylated maternally. DMR1 up-regulates the expression of H19/IGF2 and KvDMR1 lies in the promoter region of the non-coding KCNQ1OT1. KCNQ1OT1 is paternally expressed and is believed to down-regulate the expression of a number of maternally expressed genes [24]. Knockdown studies of KCNQ1OT1 showed inhibition of the cell proliferation, migration, invasion and down-regulation of drug-resistant genes including ATP binding cassette subfamily C member 5 (MRP5), multi-drug resistance mutation 1 (MDR1), and low-density lipoprotein receptor-related protein 1 (LRP1). In addition, bioinformatics analysis and also dual-luciferase reporter assay revealed that KCNQ1OT1 controlled the expression of ATP binding cassette subfamily C member 1 (ABCC1) via endogenous sponging miR-7-5p by directly targeting the 3’-UTR of miR-7-5p. Conclusively, KCNQ1OT1 similarly exerted positive effect in the treatment of oxaliplatin resistance in liver cancer via miR-7-5p/ ABCC1 axis, offering a unique strategy for the hepatocellular cancer therapy. In this study, we performed TUNEL to evaluate the apoptosis of cancer tissue from the four groups. The apoptosis of the tissue samples was gradually decreased in A, B, C and D groups.

The KCNQ1OT1 has been shown to be carcinogenic in several tumors including melanoma, hepatocellular carcinoma (HCC), glioma, and so forth by previous reports [25, 26]. KCNQ1OT1 is also associated with chemotherapy resistance and acts as a lung adenocarcinoma oncogene [27]. Our current studies conclude that the KCNQ1OT1 modifies L-OHP resistance through miR-7- 5p/ABCC1 axis in HCC, thus promising to be a groundbreaking therapy for HCC. And the knockdown of KCNQ1OT1 reduced the cell stability and increased the apoptosis rates upon L-OHP treatment. In our research, we discovered that levels of KCNQ1OT1 expression were associated with the prognosis of colon cancer patients. Subsequently, we show for the first time that miR-34a is a target of KCNQ1OT1, whereas KCNQ1OT1 might partially make colon cancer cells more vulnerable to L-OHP treatment by inhibiting protective autophagy and boosting apoptosis.

Meanwhile, a novel target of KCNQ1OT1, miR-34a, has been reported in this study. The expression of miRNA is often dysregulated in tumor cells and miRNAs are well-known to control both progression of cell-cycle as well as apoptosis. Further we noted here that tumor protein p53 targets miR-34a. We show that significant global changes in gene expression and increased apoptosis was produced by this miRNA. The transcripts that influence various essential cellular processes are highly enriched among the miR-34a-responsive genes. A significant amount of evidence reveals that the reduction in miR-34a expression in PCa is related to the drug resistance and autophagy [28–30]. In cancer cells overexpressing the miR-34a show reduced expression of autophagy-related proteins, such as ATG4B, Beclin-1 and LC3B II/I in prostate cancer cells by p-AMPK down-regulation as well as up-regulation of p-mTOR. Colon cancer cells become partially sensitized towards L-OHP therapy as KCNQ1OT1 hinders protective autophagy as well as increases apoptosis. Overall, we successfully report here that a lncRNA called KCNQ1OT1 directly targets and sponges miR-34a, thus reducing the miR-34a levels as well as amplifying the sensitivities of CRC to L-OHP chemotherapy. Here, we carried out luciferase assay to investigate the down-regulating function of miR-34a on KCNQ1OT1 and ATG4B. The luciferase activities of KCNQ1OT1 and ATG4B were apparently repressed by miR-34a in HCT116 and SW480 cells. The miR-34a has emerged as a groundbreaking treatment for multidrug resistant colon cancer [31]. Our studies reported here reveal that miR-34a mimic transection which could increase efficacy of L-OHP towards CRC. lncRNA KCNQ1OT1 sponges miR-34a which promotes the chemoresistance of CRC, therefore up-regulating the expressions of ATG4B and increasing autophagy. The interaction between a lncRNA and a miRNA is usually considered bidirectional. The lncRNA and the miRNA bind and sponge with each other, resulting in a reduction of the level of both lncRNA and miRNA. Meanwhile, the stability of lncRNA is higher than that of miRNA in most of cases. Therefore, the effect of lncRNA on miRNA and the effect of miRNA on lncRNA are not always equal. The luciferase reporter system mimics the interaction of lncRNA and miRNA under a well-controlled circumstance, and the result of luciferase assay sometimes does not reflect everything about the interaction in real but only indicates a change trend. Actually, the result of the interaction between lncRNA KCNQ1OT1 and miR-34a in colon cancer [19] also showed the transfection of miR-34a reduced luciferase activity of wild-type KCNQ1OT1, but not the mutant one. This is consistent with the result of this present study.

A miR-34C-5p down-regulation was triggered by Tamm–Horsfall protein (THP), which was related to the ATG4B up-regulation and autophagy induction. Overexpression of miR-34C-5p lowered the ATG4B level as well as undermined autophagy. It also led to increase in dead cells and apoptosis in cervical cancer cells that were treated with THP. In this study, we transfected HCT116 and SW480 cells with KCNQ1OT1 siRNA and pcDNA-ATG4B. KCNQ1OT1 siRNA significantly suppressed the expression of KCNQ1OT1/ATG4B and activated the expression of miR-34a. PcDNA-ATG4B remarkably restored the KCNQ1OT1 siRNA involve cell viability loss and apoptosis increase of HCT116 and SW480 cells.

Autophagy, a highly preserved, systematic catabolic process, process is crucial in cancer cells [32, 33]. ATG4B, which controls the maturation of autophagosome, is a highly interesting target. Current research articles show direct relations between the expression of miRNAs and ATG-modulated autophagic processes. Additionally, ATG4B is involved in development of chemoresistance in numerous cancer types. The ATG4B is an important factor in the chemotherapy resistance of lung cancer, breast cancer and prostate cancer [34, 35]. In our research study, we provide further evidence that knockdown of ATG4B attenuates L-OHP-induced protective autophagy. miR-34a could attach to the 3’ -UTR site of ATG4B, thus boosting the sensitivity of L-OHP resistant CRC.

This study provided the further insight into the molecular mechanism underlying the resistance to L-HOP and the polymorphism and methylation of KCNNQ1OT1 could be used as biomarker to predict the efficacy of chemotherapy.

## CONCLUSIONS

In summary, by investigating the patients grouped according to their allele type of KCNQ1OT1 and methylation status of KCNQ1OT1 promoter, we concluded that the drug resistance in the treatment of colon cancer is most promoted in patients not carrying allele 10 and non-methylated in KCNQ1OT1 promoter. This function is accomplished by the signaling pathway of KCNQ1OT1/miR-34a/ATG4B.
